# Supplementary material for: Rapid respiratory cryptococcosis detection using targeted next-generation sequencing
Source: PLoS Negl Trop Dis. 2025 Nov 17;19(11):e0013744. doi: 10.1371/journal.pntd.0013744 (PMC12633896; doi:10.1371/journal.pntd.0013744)
Supplement: S1 Data — Section A. Pathogens that can be detected by targeted next-generation sequencing (tNGS) in this study. Table A. Clinical and laboratory findings of enrolled patients in this study. (DOCX) [file pntd.0013744.s004.docx]

**Supplementary Information**

**Section A. Pathogens that can be detected by targeted next-generation sequencing (tNGS) in this study.**

The throat swab (TS), sputum and bronchoalveolar lavage (BAL) samples were sent to a commercial company for targeted next-generation sequencing (tNGS) analysis for respiratory tract pathogens and drug-resistance genes (Respiratory 100 panel). It achieves an early diagnosis of respiratory infection by detecting 198 pathogens including 80 bacteria (42 gram-positive bacteria, including *Corynebacterium diphtheriae*, *Mycobacterium* species, *Nocardia* species, *Parvimonas micra*, *Rhodococcus* species, *Staphylococcus aureus*, *Streptococcus* species, *Tropheryma whipplei* and *Trueperella pyogenes*; 38 gram-negative bacteria, including *Acinetobacter* species, *Bacteroides fragilis*, *Bordetella* species, *Brucella* species, *Burkholderia* species, *Elizabethkingia* species*, Enterobacter cloacae* complex, *Escherichia coli*, *Fusobacterium* species, *Haemophilus influenzae*, *Klebsiella* species, *Legionella* species, *Moraxella catarrhalis*, *Neisseria meningitidis*, *Pasteurella multocida*, *Proteus mirabilis*, *Pseudomonas aeruginosa*, *Serratia marcescens* and *Stenotrophomonas maltophilia*), 79 viruses (35 DNA viruses, including BK polyomavirus, JC polyomavirus, WU polyomavirus, Herpesviridae, human bocavirus, human mastadenovirus and human parvovirus B19; and 44 RNA viruses including coxsackievirus, enterovirus, human coronavirus 229E, HKU1, NL63, OC43, human metapneumovirus, human orthobulavirus, human respirovirus, human respiratory syncytial virus, influenza A, B, C, measles morbillivirus, mumps orthorubulavirus, rhinovirus, rubella virus and SARS-CoV-2), 32 fungi (including *Aspergillus* species, *Candida* species, *Cryptococcus* species, *Fusarium* species, *Histoplasma capsulatum*, *Lichtheimia* species, *Lomentospora prolificans*, *Meyerozyma guilliermondii*, *Mucor* species, *Nakaseomyces glabrata*, *Pichia kudriavzevii*, *Pneumocystis jirovecii*, *Rhizomucor* species, *Rhizopus* species, *Scedosporium* species, *Talaromyces marneffei* and *Trichosporon asahii*), 7 mycoplasmas/chlamydia including *Chlamydia pneumoniae*, *Chlamydia psittaci*, *Chlamydia trachomatis*, *Coxiella burnetii*, *Mycoplasma pneumoniae*, *Ureaplasma parvum* and *Ureaplasma urealyticum*, and 3 kinds of antimicrobial-resistant organisms at 15 genes (carbapenem-resistant Enterobacterales, methicillin-resistant *Staphylococcus aureus* and macrolide-resistant *M. pneumoniae* at 4 mutation points of 23S rRNA) according to the manufacturer’s information.

| **List of pathogens detected using tNGS** | |  |  |
| --- | --- | --- | --- |
|  |  |  |  |
| **Pathogen types** | **Pathogens** | | |
| DNA viruses | Herpes simplex virus 1 | Herpes simplex virus 2 | Varicella Zoster Virus |
|  | Epstein-Barr virus | Cytomegalo Virus | Human herpes virus-6 |
|  | Human herpes virus-6A | Human herpes virus-6B | Human herpes virus-7 |
|  | Human mastadenovirus | Human mastadenovirus B | Human mastadenovirus C |
|  | Human mastadenovirus D | Human adenovirus 1 | Human adenovirus 2 |
|  | Human adenovirus B3 | Human adenovirus E4 | Human adenovirus 5 |
|  | Human adenovirus 6 | Human adenovirus 7 | Human adenovirus 11 |
|  | Human adenovirus 14 | Human adenovirus 21 | Human adenovirus 34 |
|  | Human adenovirus 35 | Human adenovirus 55 | Human adenovirus 57 |
|  | Human bocavirus 1 | Human bocavirus 2 | Human bocavirus 3 |
|  | Human bocavirus 4 | Human parvovirus B19 | BK polyomavirus |
|  | JC polyomavirus | WU Polyomavirus |  |
| RNA viruses | Influenza A virus | Influenza A(H1N1) virus | Influenza A(H1N1)pdm09 virus |
|  | Influenza A(H3N2) virus | Influenza A(H5N1) virus | Influenza A(H7N9) virus |
|  | Influenza B virus | Influenza B virus (B/Victoria) | Influenza B virus (B/Yamagata) |
|  | Influenza C virus | Rhinovirus | Rhinovirus A |
|  | Rhinovirus B | Rhinovirus C | Human respiratory syncytial virus A |
|  | Human respiratory syncytial virus B | Mumps orthorubulavirus | Measles morbillivirus |
|  | Rubella virus | Human respirovirus 1 | Human orthorubulavirus 2 |
|  | Human respirovirus 3 | Human orthorubulavirus 4 | SARS-CoV-2 |
|  | Human coronavirus 229E | Human coronavirus HKU1 | Human coronavirus NL63 |
|  | Human coronavirus OC43 | Human metapneumovirus | Enterovirus |
|  | Enterovirus A | Enterovirus B | Enterovirus C |
|  | Enterovirus D | Enterovirus A71 | Coxsackievirus A2 |
|  | Coxsackievirus B3 | Coxsackievirus A5 | Coxsackievirus A6 |
|  | Coxsackievirus A10 | Coxsackievirus A16 | Echovirus E18 |
|  | Echovirus E30 | Enterovirus D68 |  |
| Gram positive bacteria | non-tuberculous mycobacteria | *Mycobacterium avium complex* | *Mycobacterium avium* |
|  | *Mycobacterium intracellulare* | *Mycobacteroides abscessus complex* | *Mycobacteroides chelonae* |
|  | *Mycobacteroides abscessus* | *Mycobacterium xenopi* | *Mycobacterium gordonae* |
|  | *Mycobacterium kansasii* | *Mycobacterium scrofulaceum* | *Mycobacterium malmoense* |
|  | *Mycobacterium shimoidei* | *Mycobacterium szulgai* | *Mycobacterium asiaticum* |
|  | *Mycobacterium celatum* | *Mycobacterium simiae* | *Mycolicibacterium smegmatis* |
|  | *Mycolicibacterium fortuitum* | *Mycobacterium tuberculosis complex* | *Nocardia* |
|  | *Nocardia concava* | *Nocardia brasiliensis* | *Nocardia farcinica* |
|  | *Nocardia africana* | *Nocardia abscessus* | *Nocardia cyriacigeorgica* |
|  | *Nocardia terpenica* | *Nocardia otitidiscaviarum* | *Nocardia nova* |
|  | *Nocardia asteroides* | *Parvimonas micra* | *Rhodococcus hoagii* |
|  | *Staphylococcus aureus* | *Streptococcus pneumoniae* | *Streptococcus pyogenes* |
|  | *Streptococcus agalactiae* | *Streptococcus anginosus group* | *Streptococcus intermedius* |
|  | *Trueperella pyogenes* | *Tropheryma whipplei* | *Corynebacterium diphtheriae* |
| Gram negative bacteria | *Acinetobacter baumannii* | *Haemophilus influenzae* | *Moraxella catarrhalis* |
|  | *Acinetobacter junii* | *Legionella* | *Bordetella pertussis* |
|  | *Acinetobacter ursingii* | *Legionella pneumophila* | *Bordetella parapertussis* |
|  | *Enterobacter cloacae complex* | *Legionella longbeachae* | *Bordetella holmesii* |
|  | *Escherichia coli* | *Legionella bozemanae* | *Elizabethkingia anophelis* |
|  | *Klebsiella pneumoniae* | *Legionella micdadei* | *Elizabethkingia meningoseptica* |
|  | *Klebsiella variicola* | *Burkholderia mallei* | *Brucella* |
|  | *Klebsiella aerogenes* | *Burkholderia pseudomallei* | *Neisseria meningitidis* |
|  | *Klebsiella oxytoca* | *Burkholderia cepacia complex* | *Pasteurella multocida* |
|  | *Proteus mirabilis* | *Burkholderia cepacia* | *Bacteroides fragilis* |
|  | *Pseudomonas aeruginosa* | *Burkholderia cenocepacia* | *Fusobacterium nucleatum* |
|  | *Serratia marcescens* | *Burkholderia contaminans* | *Fusobacterium necrophorum* |
|  | *Stenotrophomonas maltophilia* | *Burkholderia multivorans* |  |
| Fungi | *Aspergillus fumigatus* | *Rhizopus* | *Candida albicans* |
|  | *Aspergillus flavus complex* | *Rhizopus oryzae* | *Candida tropicalis* |
|  | *Aspergillus niger complex* | *Rhizopus delemar* | *Candida parapsilosis* |
|  | *Aspergillus terreus complex* | *Rhizopus microsporus* | *Candida orthopsilosis* |
|  | *Cryptococcus neoformans* | *Rhizomucor* | *[Candida] glabrata* |
|  | *Cryptococcus gattii* | *Rhizomucor pusillus* | *Pichia kudriavzevii* |
|  | *Pneumocystis jirovecii* | *Mucor irregularis* | *Trichosporon asahii* |
|  | *Talaromyces marneffei* | *Mucor racemosus* | *Meyerozyma guilliermondii* |
|  | *Lichtheimia* | *Scedosporium* | *Histoplasma capsulatum* |
|  | *Lichtheimia ramosa* | *Scedosporium apiospermum* | *Fusarium* |
|  | *Lichtheimia corymbifera* | *Scedosporium boydii* |  |
| *Mycoplasma, Chlamydia,* etc | *Mycoplasma pneumoniae* | *Chlamydia pneumoniae* | *Coxiella burnetii* |
|  | *Ureaplasma parvum* | *Chlamydia trachomatis* |  |
|  | *Ureaplasma urealyticum* | *Chlamydia psittaci* |  |

**Table A: Clinical and laboratory findings of enrolled patients in this study.**

| Patient ID | Patient history | Immunocompromised | Respiratory specimen for tNGS | Microbes detected by tNGS (sequence reads) | Pathogen identified by fungal culture and(or) histopathology | Serum CrAg |
| --- | --- | --- | --- | --- | --- | --- |
| P1 | 30 yo male with lung nodules | No | BAL | *Cryptococcus neoformans* (54129) | Negative | Positive |
| P2 | 31 yo male with lung nodules | No | BAL | *Cryptococcus neoformans* (89),  *Tropheryma whipplei* (453) | Negative | Positive |
| P3 | 66 yo female with SLE and diabetes | Yes | BAL | *Cryptococcus neoformans* (1), *Stenotrophomonas maltophilia* (5750), *Tropheryma whipplei* (399) | Negative | Positive |
| P4 | 36 yo female with SLE | Yes | BAL | *Cryptococcus neoformans* (4045), *Fusobacterium nucleatum* (2577)*, Streptococcus anginosus group* (163)*, Candida albicans* (195)*,*  Epstein-Barr virus (669) | Negative | Positive |
| P5 | 42 yo male with lung nodules | No | BAL | *Cryptococcus neoformans* (27230),  *Tropheryma whipplei* (252) | *Cryptococcus neoformans* | Positive |
| P6 | 46 yo male with pneumonia | No | BAL | *Cryptococcus neoformans* (633) | Negative | Positive |
| P7 | 34 yo male living with HIV | Yes | BAL | *Cryptococcus neoformans* (350)*, Streptococcus anginosus* (2502), *Tropheryma whipplei* (2044), *Staphylococcus aureus* (919), *Parvimonas micra* (94), *Fusobacterium nucleatum* (958), *Pneumocystis jirovecii* (26728), *Candida albicans* (24204),  Epstein-Barr virus (7214), Cytomegalovirus (37) | *Candida albicans* | Positive |
| P8 | 37 yo female with diabetes | Yes | BAL | *Cryptococcus neoformans* (30991) | *Cryptococcus neoformans* | Positive |
| P9 | 83 yo female with myasthenia gravis and diabetes | Yes | Sputum | *Cryptococcus neoformans* (6317), *Streptococcus anginosus* (246), *Fusobacterium nucleatum* (50), *Candida albicans* (36743), *Pneumocystis jirpvecii* (2880), Cytomegalovirus (313), Epstein-Barr virus (16668), Human herpesvirus-6 (18) | *Candida albicans* | Positive |
| P10 | 76 yo female with lung nodules | No | BAL | *Cryptococcus neoformans* (1775) | Negative | Positive |
| P11 | 64 yo male with lung nodules | No | BAL | *Cryptococcus neoformans* (738), *Parvimonas micra* (3159), *Fusobacterium nucleatum* (1896), *Streptococcus anginosus* (493),  Herpes simplex virus-1 (15) | Negative | Positive |
| P12 | 54 yo female with hematologic malignancy | Yes | Sputum | *Fusobacterium nucleatum* (117), EBV (2075) | Negative | Positive |
| P13 | 38 yo female with lung nodules | No | BAL | *Cryptococcus neoformans* (9035) | Negative | Positive |
| P14 | 94 yo female suspected CNS infection | No | BAL | *Pseudomonas aeruginosa* (19973), *Enterobacter cloacae complex* (110), *Acinetobacter baumannii* (246), *Stenotrophomonas maltophilia* (177), *Candida albican* (361), HSV-1 (7589), Human parvovirus B 19 (94), CMV (993), EBV (4785) | *Cryptococcus neoformans* | Positive |
| P15 | 41 yo male with lung nodules | No | BAL | *Cryptococcus neoformans* (58973) | Culture negative; Histopathology: Fungal clusters were visible | Positive |
| P16 | 62 yo male with myasthenia gravis | Yes | BAL | *Cryptococcus neoformans* (27676), *Haemophilus influenzae* (39312), *Staphylococcus aureus* (1099), Cytomegalovirus (4789), Epstein-Barr virus (9898) | *Cryptococcus neoformans* | Positive |
| P17 | 41 yo female with lung nodules | No | BAL | *Cryptococcus neoformans* (88), *Haemophilus influenzae* (175), *Staphylococcus aureus* (21), *Fusobacterium nucleatum* (103), *Streptococcus intermedius* (38), *Pneumocystis jirovecii* (109) | Negative | Positive |
| P18 | 66 yo male with diabetes | Yes | BAL | *Cryptococcus neoformans* (83013),  *Pneumocystis jirovecii* (698) | *Cryptococcus neoformans* | Positive |
| P19 | 55 yo female with cancer | Yes | BAL | *Staphylococcus aureus* (33) | Negative | Positive |
| P20 | 68 yo male with diabetes | Yes | BAL | *Cryptococcus neoformans* (73023),  *Pneumocystis jirovecii* (796) | Negative | Positive |
| P21 | 75 yo female with pneumonia | No | BAL | *Cryptococcus neoformans* (962),  *Pneumocystis jirovecii* (100),  Epstein-Barr virus (70) | Negative | Positive |
| P22 | 91 yo female with liver cancer | Yes | Sputum | *Cryptococcus neoformans* (48), *Candida albican* (1076), *Haemophilus influenzae* (28526), *Streptococcus pneumoniae* (6029), *Acinetobacter baumannii* (1254), herpes simplex virus-1 (4353), Epstein-Barr virus (680) | Negative | Not check |
| P23 | 69 yo male with pneumonia | No | BAL | *Cryptococcus neoformans* (368), *Staphylococcus aureus* (1452), *Klebsiella pneumoniae* (380), *Candida albicans* (6954),  Human herpesvirus-6 (877) | *Candida glabrata* | Not check |
| P24 | 80 yo male with pneumonia | No | BAL | *Cryptococcus neoformans* (553), *Klebsiella aerogenes* (9668), *ccus faecalis* (6150) | Negative | Not check |
| P25 | 75 yo male with prostatic cancer | Yes | BAL | *Cryptococcus neoformans* (30), *Pseudomonas aeruginosa* (899), *Candida albicans* (22707), *Pneumocystis jirovecii* (148), Herpes simplex virus-1 (3277), Epstein-Barr virus (726),  Human metapneumovirus (46032) | *Candida albicans*, Candida glabrata | Negative |
| P26 | 89 yo male with prostatic cancer | Yes | BAL | *Cryptococcus neoformans* (828), *Staphylococcus aureus* (1178), *Acinetobacter baumannii* (42341), *Klebsiella pneumoniae* (19571), Herpes simplex virus-1 (477) | Negative | Negative |
| P27 | 83 yo female with RA | Yes | Sputum | *Cryptococcus neoformans* (189), *Staphylococcus aureus* (266), *Streptococcus anginosus* (9253), *Candida albicans* (5788), SARS-CoV-2 (36130) | Negative | Negative |
| P28 | 84 yo male with diabetes | Yes | Sputum | *Cryptococcus neoformans* (25225), *Acinetobacter baumannii* (819), *Streptococcus anginosus* (1270), *Fusobacterium nucleatum* (847), *Candida albicans* (12415), SARS-CoV-2 (19947),  Epstein-Barr virus (28) | Negative | Negative |
| P29 | 83 yo male with prostatic cancer and diabetes | Yes | BAL | *Cryptococcus neoformans* (14541), *Candida tropicalis* (9531), Herpes simplex virus-1 (4276),  Epstein-Barr virus (50) | *Cryptococcus neoformans* | Negative |
| P30 | 84 yo male with prostatic cancer and diabetes | Yes | BAL | *Cryptococcus neoformans* (12281), *Candida albicans* (7994), *Pneumocystis jirovecii* (2557),  Herpes simplex virus-1 (244),  Epstein-Barr virus (181) | negative | Negative |
| P31 | 83 yo male with pneumonia | No | BAL | *Cryptococcus neoformans* (124), *Klebsiella pneumoniae* (43362), *Escherichia coli* (19974), *Staphylococcus aureus* (14395), *Acinetobacter baumannii* (10409), *Streptococcus anginosus* (3025), Epstein-Barr virus (91) | *Candida parapsilosis complex* | Not check |
| P32 | 82 yo male with colon cancer | Yes | BAL | *Cryptococcus neoformans* (590), *Escherichia coli* (35254), SARS-CoV-2 (30587) | *Candida glabrata* | Negative |
| P33 | 87 yo male with diabetes | Yes | BAL | *Cryptococcus neoformans* (554), *Klebsiella pneumoniae* (29043), *Aspergillus fumigatus* (3433), *Candida albicans* (1945), Herpes simplex virus-1 (21705), Epstein-Barr virus (62) | *Candida albicans* | Negative |
| P34 | 84 yo male with osteosarcoma and diabetes | Yes | Sputum | *Cryptococcus neoformans* (1940), *Staphylococcus aureus* (2073), *Streptococcus agalactiae* (168), *Klebsiella pneumoniae* (53), *Acinetobacter baumannii* (507), *Streptococcus anginosus* (1325), *Parvimonas micra* (192), *Candida albicans* (2263), *Pneumocystis jirovecii* (48), Epstein-Barr virus (1104), Human herpesvirus-7 (29) | Negative | Not check |
| P35 | 74 yo male with carcinoma of ampulla and RA | Yes | BAL | *Cryptococcus neoformans* (1139),  *Candida albicans* (11622), Epstein-Barr virus (276) | *Candida glabrata* | Negative |
| P36 | 51 yo male with lung cancer | Yes | Sputum | *Cryptococcus neoformans* (46257), *Staphylococcus aureus* (1740), *Streptococcus mitis* (229), *Fusobacterium nucleatum* (43),  Human herpesvirus-7 (137) | *Cryptococcus neoformans* | Negative |
| P37 | 29 yo male with lung nodules | No | BAL | *Cryptococcus neoformans* (71592),  *Pneumocystis jirovecii* (209) | *Cryptococcus neoformans* | Positive |
| P38 | 74 yo male with lung cancer | Yes | BAL | *Cryptococcus neoformans* (229),  *Pseudomonas aeruginosa* (34256),  Human herpesvirus-7 (42) | *Cryptococcus neoformans* | Negative |
| P39 | 85 yo male with antiphospholipid syndrome | Yes | BAL | *Cryptococcus neoformans* (1113),  *Candida albicans* (50989), Epstein-Barr virus (65) | *Candida albicans* | Negative |

**Remarks:** tNGS: target-Next Generation Sequencing; CrAg: Cryptococcal antigen; BAL: bronchoalveolar lavage; SLE: Systemic Lupus Erythematosus; RA: Rheumatoid arthritis.
