# Supplementary material for: Rapid respiratory cryptococcosis detection using targeted next-generation sequencing
Source: PLoS Negl Trop Dis. 2025 Nov 17;19(11):e0013744. doi: 10.1371/journal.pntd.0013744 (PMC12633896; doi:10.1371/journal.pntd.0013744)
Supplement: Attachment — Submitted filename: Reviewer_Comments_tNGS_Manuscript.docx [file pntd.0013744.s005.docx]

# Comments

## DNA Quality and Host Background

The manuscript would benefit from clearer methodological details on the quality and quantity of DNA used for tNGS. Although the extraction protocol is described (lines 142–150), the authors do not specify DNA input concentrations or purity measures (e.g., Qubit quantification, A260/280 ratios, or minimum DNA mass per library).
Because host-derived nucleic acids typically dominate respiratory specimens, the ratio of human to microbial DNA is a critical factor influencing tNGS sensitivity and pathogen read depth. To improve reproducibility and technical transparency, the authors should:
• Report the minimum DNA input required for library preparation and the average DNA yield per sample.
• Clarify whether any host DNA depletion or microbial enrichment steps were performed before sequencing.
• Discuss whether excessive host DNA background could explain the low microbial read counts or false-negative tNGS results observed in some cases.

## Ethical and Data Transparency

The study is retrospective and appropriately approved by an ethics committee. However:
• The data availability statement should clearly indicate whether raw sequencing data (FASTQ files or summary read counts) are deposited in a public repository, rather than stating they are available “upon reasonable request.”
• The authors should clarify whether informed consent for secondary use of clinical data was obtained, or if this requirement was waived under institutional review board approval.

## Statistical and Methodological Clarifications

• The manuscript lacks a power calculation or justification for the relatively small sample size (n = 39). While this limitation is acknowledged, presenting confidence intervals would help convey the precision of the findings.
• The authors should specify the exact statistical test used to compare diagnostic yields (e.g., Chi-square or Fisher’s exact test).
• For turnaround time comparisons, the median and interquartile range (IQR) values should be explicitly reported in the Results section, not only in figure captions.

## Cost-Effectiveness and Accessibility

The authors are encouraged to include a brief discussion on the economic feasibility of tNGS compared with existing diagnostic methods. Specifically:
• Provide an approximate per-sample cost for tNGS (including sequencing and analysis) versus CrAg testing and fungal culture.
• Discuss opportunities to reduce costs through batching or multiplexing.
• Address the trade-off between higher diagnostic yield and higher per-test cost.
• Comment on whether faster turnaround and earlier treatment initiation could offset laboratory expenses, thereby improving overall cost-effectiveness.

## Language and Style Improvements

Overall, the manuscript is clear but would benefit from professional language editing to improve conciseness and flow. Below are selected examples:

**Abstract**
• “Polymicrobial were detected in 76.9% (30/39) of tNGS testing…” → “Polymicrobial infections were detected in 76.9% (30/39) of tNGS tests.”

**Author Summary**
• “Results from tNGS were drastically faster than the fungal culture.” → “tNGS results were available much faster than fungal culture.”
• “We propose integrating tNGS…” → “We recommend integrating tNGS…”

**Introduction**
• “Current clinical diagnosis… each with notable limitations.”
• “Consequently, diagnostic delays often lead to meningitis and increased mortality.”

**Materials and Methods**
• “This study was conducted over a 38 months period…” → “This study was conducted over a 38-month period.”
• “Clinical specimens were collected and handled according to standard protocols 16.” → “Clinical specimens were collected and processed following standard protocols [16].”
• “Qualified lower respiratory samples (≥600 μL)… transported…” → “Lower respiratory samples (≥600 μL) were collected in sterile tubes, stored at 4 °C, and transported within 8 hours for tNGS analysis.”

**Discussion**
• “Retrospective analysis of turnaround times…” → “Turnaround time analysis showed that tNGS identified C. neoformans significantly faster than culture (median 23 h vs. 112 h; P < 0.05).”
• “…respiratory specimens from 3 patients…” → “…three patients with pulmonary cryptococcosis tested negative for C. neoformans by tNGS (patients 12, 14, and 19).”

**Conclusion**
• “Our study highlights the high sensitivity…” → “This study demonstrates that tNGS provides high sensitivity and markedly shorter turnaround time compared with conventional culture.”

**General Notes**
• Avoid redundant phrases such as “as previously mentioned.”
• Use active voice for clarity.
• Maintain consistent tense and use formal transition words (e.g., “Moreover,” “Therefore”).
