# Supplementary material for: Rapid respiratory cryptococcosis detection using targeted next-generation sequencing
Source: PLoS Negl Trop Dis. 2025 Nov 17;19(11):e0013744. doi: 10.1371/journal.pntd.0013744 (PMC12633896; doi:10.1371/journal.pntd.0013744)
Supplement: Attachment — Submitted filename: Response to Reviewers.docx [file pntd.0013744.s006.docx]

Dear editor and reviewers:

Thank you for your decision and the constructive comments on our manuscript. We greatly appreciate your comments and suggestions!

Below, you will find our itemized responses, along with the corrections made in the resubmitted files. Additionally, the revisions are highlighted in yellow for your convenience.

# Comments

## DNA Quality and Host Background

The manuscript would benefit from clearer methodological details on the quality and quantity of DNA used for tNGS. Although the extraction protocol is described (lines 142–150), the authors do not specify DNA input concentrations or purity measures (e.g., Qubit quantification, A260/280 ratios, or minimum DNA mass per library).
Because host-derived nucleic acids typically dominate respiratory specimens, the ratio of human to microbial DNA is a critical factor influencing tNGS sensitivity and pathogen read depth. To improve reproducibility and technical transparency, the authors should:
• Report the minimum DNA input required for library preparation and the average DNA yield per sample.
• Clarify whether any host DNA depletion or microbial enrichment steps were performed before sequencing.
• Discuss whether excessive host DNA background could explain the low microbial read counts or false-negative tNGS results observed in some cases.

**Response:** We sincerely thank the reviewer for raising this important point regarding DNA quality and the potential impact of host nucleic acids. We agree that technical transparency is crucial for reproducibility. The core of our method is multiplex PCR-based target enrichment NGS, which fundamentally differs from shotgun metagenomics. In our protocol, cDNA/DNA is used as a template for a multiplex PCR reaction that uses a large panel of primers specific to the 198 targeted pathogens. Consequently, the sequencing library is constructed from these amplicons, not from randomly sheared total DNA. This means the sensitivity and pathogen read depth are primarily dependent on the successful amplification of the target loci, not on the initial ratio of microbial to human DNA. Host DNA, lacking the target sequences, is not amplified and thus does not compete for sequencing depth. Therefore, we have revised the manuscript to enhance clarity: (i) we have specified the minimum DNA input quantity (1 µg) as required by our library preparation kit (RP100™). (ii) we have explicitly stated that no host DNA depletion steps were performed. (iii) we have revised the methods section to explain this principle, clarifying that the multiplex PCR amplification negates the confounding effect of host DNA background. (L141-183)

## Ethical and Data Transparency

The study is retrospective and appropriately approved by an ethics committee. However:
• The data availability statement should clearly indicate whether raw sequencing data (FASTQ files or summary read counts) are deposited in a public repository, rather than stating they are available “upon reasonable request.”
• The authors should clarify whether informed consent for secondary use of clinical data was obtained, or if this requirement was waived under institutional review board approval.

**Response:** We thank the reviewer for these critical points regarding data availability and ethics. As the tNGS testing was performed by a third-party company (KingMed Diagnostics), the original FASTQ files are retained by them and not available for public deposition. As a solution to ensure the verifiability of our results, we have deposited the complete dataset of summary read counts as a Supplementary Table 2 accompanying the manuscript. This dataset contains all the quantitative information necessary to replicate our primary findings and analyses. The Data Availability Statement has been updated to explicitly state: "The authors confirm that all data underlying the findings are fully available without restriction. All relevant data are within the manuscript and supplementary file.”

## Statistical and Methodological Clarifications

• The manuscript lacks a power calculation or justification for the relatively small sample size (n = 39). While this limitation is acknowledged, presenting confidence intervals would help convey the precision of the findings.
• The authors should specify the exact statistical test used to compare diagnostic yields (e.g., Chi-square or Fisher’s exact test).
• For turnaround time comparisons, the median and interquartile range (IQR) values should be explicitly reported in the Results section, not only in figure captions.

**Response:** We sincerely thank the reviewer for these valuable and constructive comments. We have revised the manuscript to enhance clarity: (i) added 95% confidence intervals (CIs) for all proportional data in Table 1. (ii) explicitly specified the statistical tests used for all comparisons throughout the manuscript. (iii) explicitly reported the median and IQR values for the turnaround time comparisons directly in the main text of the Results section. (L229-230)

## Cost-Effectiveness and Accessibility

The authors are encouraged to include a brief discussion on the economic feasibility of tNGS compared with existing diagnostic methods. Specifically:
• Provide an approximate per-sample cost for tNGS (including sequencing and analysis) versus CrAg testing and fungal culture.
• Discuss opportunities to reduce costs through batching or multiplexing.
• Address the trade-off between higher diagnostic yield and higher per-test cost.
• Comment on whether faster turnaround and earlier treatment initiation could offset laboratory expenses, thereby improving overall cost-effectiveness.

**Response:** We thank the reviewer for this insightful suggestion. In response, we have incorporated a cost-feasibility discussion into the manuscript. At our institution, tNGS is priced at 750 RMB per sample—substantially higher than fungal culture (85 RMB) or CrAg testing (100 RMB). Nevertheless, this elevated cost should be interpreted in context: tNGS is amenable to batch processing, which lowers the per-test expense, and its broad pathogen detection capability can help avoid a protracted and costly diagnostic odyssey involving multiple sequential tests. Although a formal cost-effectiveness analysis lies beyond the scope of this study, we propose that the rapid turnaround time of tNGS (23 [21–43] hours) may enable earlier initiation of targeted therapy, potentially shortening hospital stays and decreasing overall healthcare expenditures. Therefore, despite its higher upfront cost, tNGS could represent a cost-effective strategy from a broader health-system perspective. (L424-433)

## Language and Style Improvements

Overall, the manuscript is clear but would benefit from professional language editing to improve conciseness and flow. Below are selected examples:
**Abstract**
• “Polymicrobial were detected in 76.9% (30/39) of tNGS testing…” → “Polymicrobial infections were detected in 76.9% (30/39) of tNGS tests.”
**Author Summary**
• “Results from tNGS were drastically faster than the fungal culture.” → “tNGS results were available much faster than fungal culture.”
• “We propose integrating tNGS…” → “We recommend integrating tNGS…”
**Introduction**
• “Current clinical diagnosis… each with notable limitations.”
• “Consequently, diagnostic delays often lead to meningitis and increased mortality.”
**Materials and Methods**
• “This study was conducted over a 38 months period…” → “This study was conducted over a 38-month period.”
• “Clinical specimens were collected and handled according to standard protocols 16.” → “Clinical specimens were collected and processed following standard protocols [16].”
• “Qualified lower respiratory samples (≥600 μL)… transported…” → “Lower respiratory samples (≥600 μL) were collected in sterile tubes, stored at 4 °C, and transported within 8 hours for tNGS analysis.”
**Discussion**
• “Retrospective analysis of turnaround times…” → “Turnaround time analysis showed that tNGS identified C. neoformans significantly faster than culture (median 23 h vs. 112 h; P < 0.05).”
• “…respiratory specimens from 3 patients…” → “…three patients with pulmonary cryptococcosis tested negative for C. neoformans by tNGS (patients 12, 14, and 19).”
**Conclusion**
• “Our study highlights the high sensitivity…” → “This study demonstrates that tNGS provides high sensitivity and markedly shorter turnaround time compared with conventional culture.”
**General Notes**
• Avoid redundant phrases such as “as previously mentioned.”
• Use active voice for clarity.
• Maintain consistent tense and use formal transition words (e.g., “Moreover,” “Therefore”).

**Response:** We thank the reviewer for their valuable suggestions, which have been incorporated into the manuscript. The text has also undergone thorough proofreading to enhance its clarity and academic tone.

**Reviewers' comments:**

Reviewer #1: (No Response)

Reviewer #2: -The objectives are generally stated but the hypothesis is not explicitly articulated. The authors should clearly define a testable hypothesis reflecting the comparative evaluation of the three diagnostic methods for cryptococcosis.

**Response:** We thank the reviewer for this valuable comments. The Introduction has been revised accordingly to provide a clearer foundation for our study. (L87-96)

-Figure 1 and study population:
Figure 1 indicates that 80 patients with confirmed cryptococcosis were enrolled, implying that the European Organization for Research and Treatment of Cancer and the Mycoses Study Group Education and Research Consortium (EORTC/MSGERC) guideline was used as the diagnostic gold standard. This should be explicitly stated in the Methods section to clarify the reference framework for case confirmation.

**Response:** We thank the reviewer for this constructive feedback. The suggested revisions have now been incorporated into the methods section. (L106-112)

While the manuscript primarily focuses on tNGS, the diagnostic performance of the other two methods evaluated is also of clinical relevance. Given that the study population (n = 39) may be insufficient to independently establish the diagnostic accuracy of tNGS, I recommend including a comparative performance analysis of all three methods using the same patient cohort. Highlighting how tNGS performs relative to established methods would enhance the clinical value and originality of the work, framing it as a true comparative diagnostic study—the main novelty identifiable from the current manuscript.

**Response:** We thank the reviewer for this valuable suggestion. To enable a direct and methodologically sound comparison, we have focused our analysis on tNGS and fungal culture, as both were performed on respiratory specimens. This approach eliminates potential spectrum bias from differing sample types (respiratory vs. serum for CrAg) and missing data. The corresponding revisions have been made in the Methods section, and the results are detailed in Table 2. (L311-325)

-The manuscript needs clarification regarding the study design. Although described as retrospective, lines 108–119 read as if the study were prospective. Please clearly state how samples were obtained and indicate whether they originated from a previous study, including its title or ethics reference if applicable. The study population is generally appropriate.

**Response:** We thank the reviewer for this important observation and apologize for the lack of clarity regarding our study design. We have now revised the Methods section to explicitly state the retrospective nature of the data and sample collection. (L106-112)

-There are inconsistencies regarding informed consent (lines 103–105 versus 186–187). Please clarify whether patient consent was obtained and confirm compliance with ethical standards.

**Respon**se: Thank you for your comment. We apologize for the confusion. While informed consent was signed by all participants prior to testing, this retrospective study meets the ethical criteria for exemption from such consent. To avoid any ambiguity in the context, we have revised this part. (L101-103 and L192-194)

•Line 146: Clarify the use of 0.1 M DTT for mucolysis, as 0.1% (w/v) is the more typical concentration. Provide a citation or indicate if this followed a manufacturer’s protocol.

**Response**: We have revised this sentence in accordance with the reviewer's suggestions. (L144)

•Line 144: Replace “commercial company laboratory” with “private laboratory” or “the laboratory of a private company.”

**Response:** Thank you for your comment. We have revised this sentence in accordance with the reviewer's suggestions. (L141-143)

**Results**
Reviewer #2: -Lines 277–280: The two sentences appear contradictory. Clarify what was actually performed.

**Response:** Thank you for this feedback. We have revised the indicated sentences to enhance their clarity and precision. (L311-313)

-The authors should report the calculated sensitivity and specificity of tNGS compared to the other diagnostic methods (or at least the gold standard) to substantiate conclusions made later in the manuscript (line 432).

Response: Thank you for your comment. We compared the diagnostic efficacy of tNGS to fungal culture; the results are presented in Table 2.(L320-326)

-Table 1: Age is expressed as years (IQR) while other variables are n (%). Find a way to indicate this.

**Response:** Thank you for your comment. We have added 95% confidence intervals for the other data. (Table 1)

-Figure 4: The flowchart is confusing. Usually, boxes containing questions and others with “yes”/“no” or “positive”/”negative” should be used to denote what decision to take after an outcome. For instance, the current chart does not indicate what happens to “respiratory specimen for culture”.

**Response:** Thank you for your comment. To make the flowchart clearer and more understandable, we have revised Figure 4.

-Line 289: The title “Clinical implication of tNGS for cryptococcosis” may be better phrased as “Diagnostic implication of tNGS for cryptococcosis.”

**Response:** Thank you for your comment. We have revised it based on the suggestions. (L284)

**Conclusions**

Reviewer #2: The conclusions that tNGS has high sensitivity and specificity should fully supported by quantitative evidence in the results by specifically calculating these.

**Response:** Thank you for your comment. We compared the diagnostic efficacy of tNGS to fungal culture; the results are presented in Table 2

**Editorial and Data Presentation Modifications?**

Reviewer #2: •Title: Does not fully reflect the manuscript’s content. Recommend revising to highlight the comparative nature of the study (e.g., “Comparative Evaluation of Three Diagnostic Methods for Cryptococcosis in a Clinical Setting”).

**Response**: We thank the reviewer for this valuable suggestion. After careful consideration, we have decided to retain the original title. We believe it accurately reflects the study's focus on demonstrating the clinical utility of tNGS for the rapid diagnosis of respiratory cryptococcosis.

• Line 66 should specify which organs are most commonly affected by cryptococcosis to provide clinical context.

**Response:** Thank you for your comment. We revised this sentence to make the expression clearer. (L63-66)
• Line 91: Define “t” in tNGS at first mention.

**Response:** Thank you for your comment. “t” has been defined accordingly. (L88)

•Line 92: Italicize all species names.

**Response:** Thank you for your comment. All species names are italicized.

•Line 237: Replace aures with aureus. Ensure subsequent mentions are abbreviated properly and italicized.

**Response:** Thank you for your comment. The species names have been revised to ensure accurate spelling. (L245)
•Lines 262–263: The sentence beginning “The other two cases…” is grammatically incorrect and confusing. Revise to clearly describe the findings.

**Response:** Thank you for your comment. We revised this sentence to make the expression clearer. (L269-271)
•Line 349: Change to “potentially increasing the sensitivity of tNGS” instead of “potentially increased tNGS sensitivity.”

**Response:** Thank you for your comment. We revised this sentence to make the expression clearer. (L361)
•Lines 385–392 provide a weak rationale for the observed limitation. In active infections, tNGS should detect genetic material if sample collection and processing are appropriate. The limitation is more likely due to sample handling, as discussed in lines 416–418. Revise the earlier explanation accordingly.

**Response:** Thank you for your comment. We have revised this part based on your suggestions, making the expression more precise.(L396-405)
•Line 394: Delete “in.”

**Response:** Thank you for your comment. We revised this sentence to make the expression clearer. (L406-408)
•Discussion: Reduce redundancy by removing repeated descriptions of the results. Focus instead on interpretation and implications.

**Response:** Thank you for your comment. We have revised this part based on your suggestions, making the expression more precise.
•Ensure all abbreviations are defined at first use and standardized throughout the manuscript.

**Response:** Thank you for your comment. All abbreviations have been defined at first use and standardized.
•Grammar and syntax should be checked carefully throughout for consistency and conciseness.

**Response:** Thank you for your comment. We have already checked and revised the grammar and syntax of the manuscript.

**Summary and General Comments**

Reviewer #2: The manuscript is clearly written with only minor errors. The objectives are generally stated but the hypothesis is not explicitly articulated. The study population is generally appropriate and the manuscript has clearly defined sample characteristics and the limitation acknowledged. The above corrections should be considered to improve the manuscript.

**Response:** We thank the reviewer for their thoughtful review and for acknowledging the strengths of our work. We have taken the feedback to heart and have revised the manuscript to include an explicit hypothesis, as recommended. All other minor corrections have been diligently addressed.

Yours sincerely,

Dr. Chaowen Deng

Department of Infectious Diseases and Microbiology

The University of Hong Kong–Shenzhen Hospital
